# Comparative Effectiveness of Oral Drug Therapies for Lower Urinary Tract Symptoms due to Benign Prostatic Hyperplasia: A Systematic Review and Network Meta-Analysis

**DOI:** 10.1371/journal.pone.0107593

**Published:** 2014-09-12

**Authors:** Xinghuan Wang, Xiao Wang, Sheng Li, Zhe Meng, Tao Liu, Xinhua Zhang

**Affiliations:** Department of Urology, Zhongnan Hospital of Wuhan University, Wuhan city, Hubei province, P.R.China; National Taiwan University, Taiwan

## Abstract

**Introduction:**

Lower urinary tract symptoms (LUTS) due to benign prostatic hyperplasia (BPH) are common in elder men and a number of drugs alone or combined are clinically used for this disorder. But available studies investigating the comparative effects of different drug therapies are limited. This study was aimed to compare the efficacy of different drug therapies for LUTS/BPH with network meta-analysis.

**Materials and Methods:**

An electronic search of PubMed, Cochrane Library and Embase was performed to identify randomized controlled trials (RCTs) comparing different drug therapies for LUTS/BPH within 24 weeks. Comparative effects were calculated using Aggregate Data Drug Information System. Consistency models of network meta-analysis were created and cumulative probability was used to rank different therapies.

**Results:**

A total 66 RCTs covering seven different therapies with 29384 participants were included. We found that α-blockers (ABs) plus phosphodiesterase 5 inhibitors (PDE5-Is) ranked highest in the test of IPSS total score, storage subscore and voiding subscore. The combination therapy of ABs plus 5α-reductase inhibitors was the best for increasing maximum urinary flow rate (Qmax) with a mean difference (MD) of 1.98 (95% CI, 1.12 to 2.86) as compared to placebo. ABs plus muscarinic receptor antagonists (MRAs) ranked secondly on the reduction of IPSS storage subscore, although monotherapies including MRAs showed no effect on this aspect. Additionally, PDE5-Is alone showed great effectiveness for LUTS/BPH except Qmax.

**Conclusions:**

Based on our novel findings, combination therapy, especially ABs plus PDE5-Is, is recommended for short-term treatment for LUTS/BPH. There was also evidence that PDE5-Is used alone was efficacious except on Qmax. Additionally, it should be cautious when using MRAs. However, further clinical studies are required for longer duration which considers more treatment outcomes such as disease progression, as well as basic research investigating mechanisms involving PDE5-Is and other pharmacologic agents alleviate the symptoms of LUTS/BPH.

## Introduction

Lower urinary tract symptoms (LUTS) secondary to benign prostatic hyperplasia (BPH) are common and interfere with the quality of life (QoL) of elder men [1]–[3]. LUTS which includes obstructive (voiding) symptoms and irritative (storage) symptoms [4] can be quantitatively evaluated by questionnaires such as the International Prostate Symptom Score (IPSS) [5]. The prevalence of BPH is approximately 40% for men in their fifties and reaches to 90% for men in their nineties [6] and the incidence of LUTS is around 25% for men in their 50 s or older [7], [8]. The drug treatment for bothersome moderate to severe LUTS/BPH aimed to relieve the symptoms and slow the clinical progression of this disease. Current oral therapies recommended by Guidelines include α-adrenoceptor antagonists (α-blockers, ABs), 5α-reductase inhibitors (5ARIs), muscarinic receptor antagonists (MRAs) and a “new emerging treatment” phosphodiesterase 5 inhibitors (PDE5-Is) [9], [10]. ABs and 5ARIs have been widely used for decades. Overactive bladder (OAB) symptoms are commonly reported by LUTS/BPH patients even post-prostatectomy [11]–[13] and MRAs have been proved efficacious in reducing bladder overactivity and storage symptoms. Recently numerous clinical trials have investigated the efficacy of PDE5-Is for LUTS/BPH, while tadalafil was recently licensed in USA and in European Union for treating LUTS/BPH with or without erectile dysfunction (ED) [9], [10]. Combining drugs from different classes had a positive synergistic effect. Common combinations include ABs plus 5ARIs, ABs plus MRAs and ABs plus PDE5-Is. Both monotherapies and combined therapies have been demonstrated efficacious for LUTS/ BPH by a large number of clinical trials worldwide. However, studies investigating the comparative effects of different types of drug therapies are limited.

The aim of our study was to carry out a systematic review and network meta-analysis comparing the efficacy of different drug therapies for LUTS/BPH based on existing randomized controlled trials (RCTs) and ranking these regimens for practical consideration.

## Materials and Methods

### Data sources and searches

We performed an electronic search of Cochrane Library, PubMed and Embase till June 2013. The search strings used for electronic searches were based on MeSH terms. Following keywords were used to search both medical subject headings terms and text words: lower urinary tract symptom *or* benign prostatic hyperplasia/enlargement *or* bladder outlet obstruction *plus* α-adrenoceptor antagonists, alfuzosin, tamsulosin, doxazosin, terazosin, naftopidil, prazosin and silodosin *or* 5α-reductase inhibitors, dutasteride and finasteride *or* muscarinic receptor antagonists, darifenacin, fesoterodin, oxybutynin, propiverine, solifenacin and tolterodine *or* phosphodiesterase 5 inhibitors, sildenafil, tadalafil, vardenafil, avanafil *plus* randomized controlled study. No limitation was placed on publication status or language.

### Selection of Studies

We included RCTs that compared different oral therapies or placebo for LUTS/BPH. The treatment duration of most trials was less than 24 weeks, especially for trials with multiple treatment arms. As trials with multiple arms are more important to build comparative loops in network meta-analysis and the consistency model of network meta-analysis required rigorous homogeneity between trials, we excluded trials with treatment duration over 24 weeks.

### Exclusion criteria

1) repeated publications; 2) studies with treatment duration longer than 24 weeks; 3) studies were not measured by the aim outcomes of IPSS score and Qmax, or the result were reported incompletely; 4) full text were unavailable or studies reported superficially, such as in the form of an abstract.

### Data extraction and quality assessment

Data were extracted independently by three reviewers (SL, ZM and TL) using a standard form. The different dosage or subgroups of one class of treatment from the original studies were pooled into one arm for analysis. Missing information was imputed based on the methods of Cochrane Handbook and when necessary, was requested from the authors of original studies. Discrepancies were resolved by discussion. The methodological quality of included studies was appraised with the Cochrane Collaboration bias appraisal tool. In particular, the following factors were evaluated: (1) Adequate sequence generation? (2) Allocation concealment? (3) Binding? (4) Incomplete outcome data addressed? (5) Free of selective reporting? (6) Free of other bias? Every question was answered with “yes”, “no” or “unclear” and three reviewers (SL, ZM and TL) assessed each trial. In case of disagreement, judgment was made through open discussion.

### Main outcome measures

The intervention outcomes were the change from baseline to study end in the IPSS (including IPSS total score, IPSS storage subscore and IPSS voiding subscore) and maximum flow rate (Qmax). Compared with pair-wise meta-analysis, network analysis can be applied in the studies with multiple treatment arms and combine both direct and indirect evidence from RCTs in order to obtain a single consistent quantitative synthesis [14]–[17]. Comparative effects of different drug treatments in the network analysis were calculated using the automated software Aggregate Data Drug Information System (ADDIS) [18]. We created a consistency model by combining the effect of indirect and direct comparison based on Bayesian approach to get an absolute effect and cumulative probability which was used to rank different drug therapies. Node splitting models were conducted to detect inconsistency in a single comparison. Direct evidence was based on pair-wise meta-analysis and indirect evidence based on indirect comparisons through the consistency models of network meta-analysis [19]. Convergence diagnostics were assessed using Brooks-Gelman-Rubin methods to determine whether the modes had converged [20]. Summary effect was calculated as mean difference (MD) for continuous variable, together with its 95% confidence intervals (CIs).

## Results

### Characteristics of included studies and quality assessment

**Figure 1:**
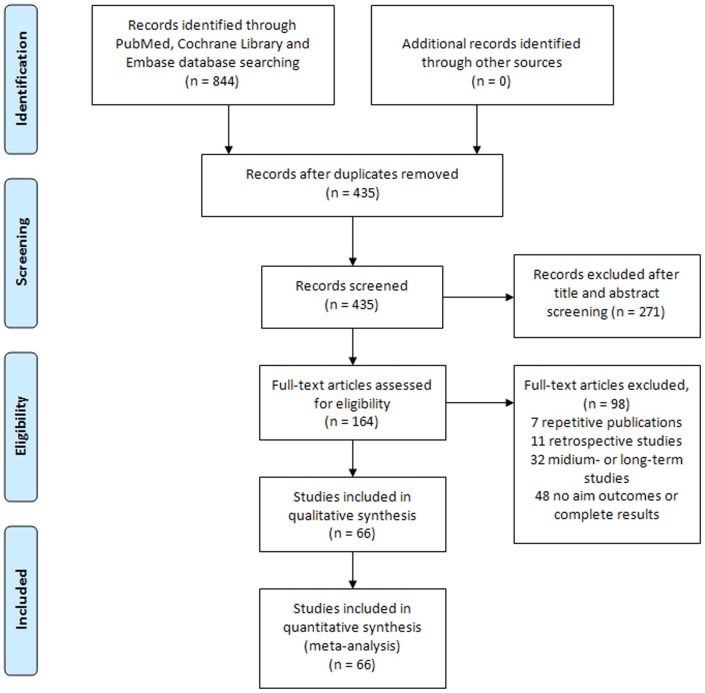
PRISMA flowchart of literature searches and results. RCT =  randomized controlled trials.

**Figure 2:**
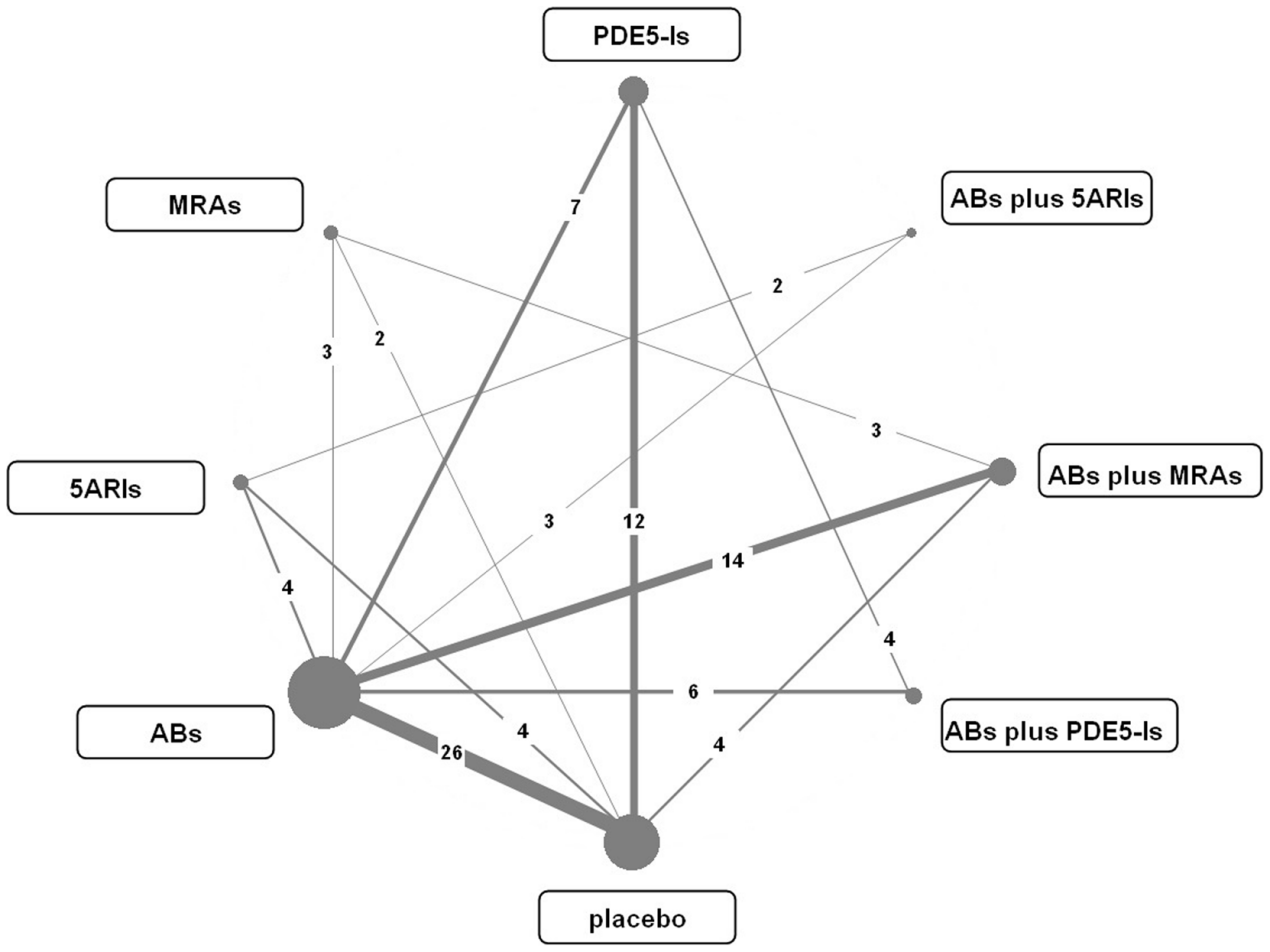
Comparison network of the included studies. The line linked between two drug therapies means there are direct comparisons from original studies. Numbers on the line mean the count number of studies comparing every pair of treatments, which were also reflected by the width of the lines. The size of every node represents the number of randomized participants. ABs  =  α-blockers. 5ARIs  =  5α-reductase inhibitors. MRAs  =  muscarinic receptor antagonists. PDE5-Is  =  phosphodiesterase 5 inhibitors.

Using the electronic search strategy, a total of 844 records were retrieved, of which 66 RCTs were finally included [21]–[86]. Fig.1 shows the flowchart of literature searches and Table S1 provides details of the included trials. The included 66 RCTs covered the currently used seven kinds of drug therapies including ABs, 5ARIs, MRAs, PDE5-Is, ABs plus 5ARIs, ABs plus MRAs and ABs plus PDE5-Is with a total 29,384 participants. Fig.2 shows the overall comparison network. The mean treatment duration of the included trials was 13.3 weeks (ranged 4 to 24 weeks). Table S2 shows summary of the risk of bias. Most studies did not provide detailed randomization and allocation methods, while five studies used inadequate randomization, thus we gave negative judgment. Blinding assessment for most included studies was judged as positive. For the assessment of incomplete outcome data, three studies reported a significant withdrawal of patients, while some others did not report withdrawal information or reasons. We gave positive judgment for all the included studies in the assessment of selective reporting and other bias, as we could not detect any risks for both aspects.

### Efficacy of treatment on included outcomes

#### IPSS total score

**Figure 3:**
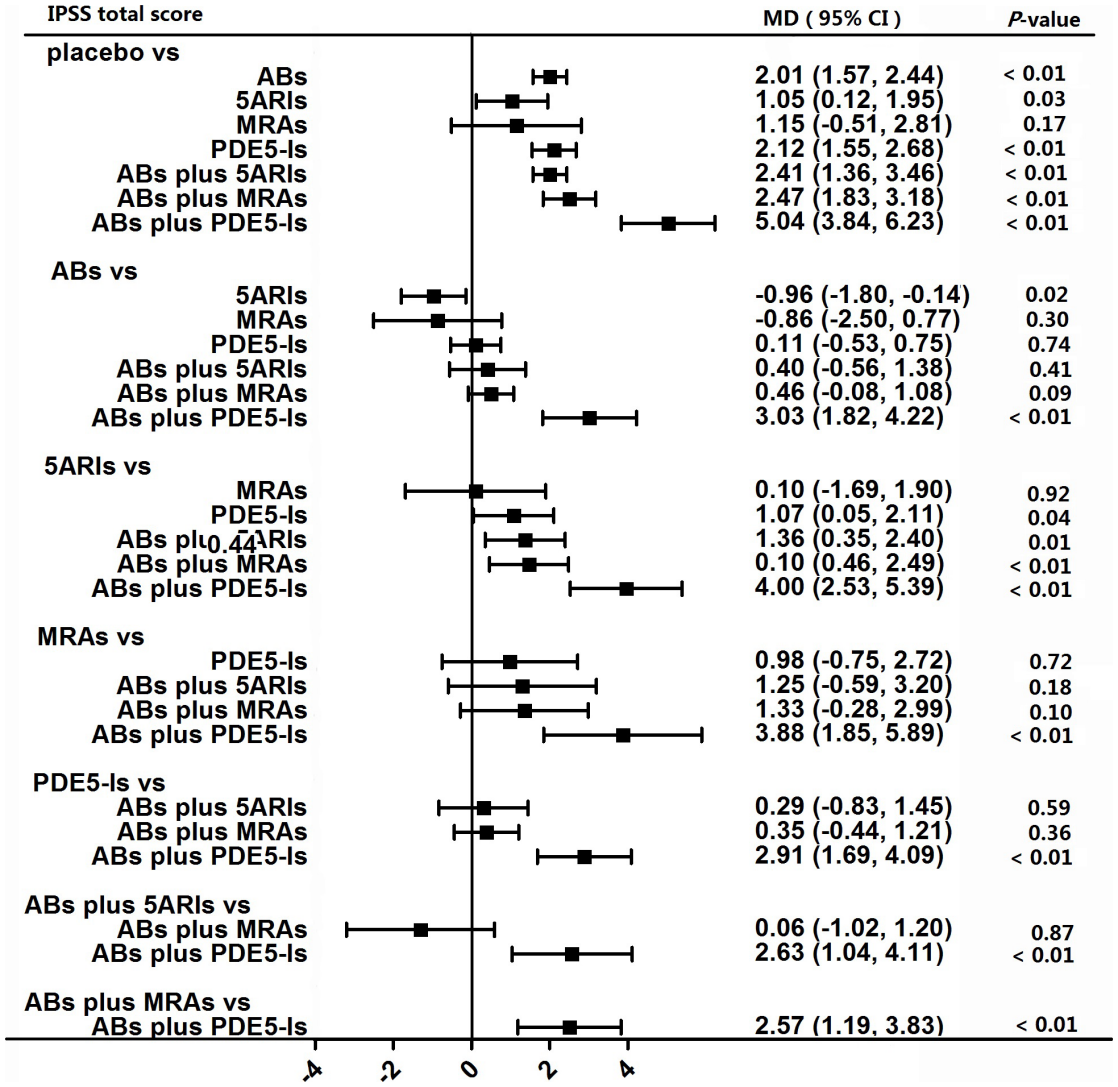
Forest plot for meta-analysis of the overall effect as measured by the IPSS total score. The difference of IPSS total score between comparisons was calculated as mean difference (MD) and MD below 0 favors the drug therapy on the left header. If the 95% confidence intervals (CI) did not include 0, it means the difference is significant. ABs  =  α-blockers. 5ARIs  =  5α-reductase inhibitors. MRAs  =  muscarinic receptor antagonists. PDE5-Is  =  phosphodiesterase 5 inhibitors.

A total of 48 studies involving all seven kinds of drug therapies contributed to the analysis of IPSS total score. Fig.3 provides the overall effect of different kinds of drugs therapies on IPSS total score. Six kinds of medical therapies had a significant effect on the reduction of IPSS total score as compared with placebo, which were ABs (p<0.01), 5ARIs (p = 0.03), PDE5-Is (p<0.01), ABs plus 5ARIs (p<0.01), ABs plus MRAs (p<0.01) and ABs plus PDE5-Is (p<0.01). Table.1 shows the results of node split models indicating that there was significant difference between direct and indirect effect in the comparisons of placebo vs ABs plus MRAs and ABs plus PDE5-Is vs ABs.

**Table 1:** Results of node split models for the test of difference between direct and indirect effect in the analysis of primary outcomes of IPSS total score, Qmax, IPSS storage subscore and IPSS voiding subscore.

Comparison	Direct Effect	Indirect Effect	Overall	P-Value
**IPSS total score**				
placebo vs ABs	1.89 (1.44, 2.34)	2.76 (1.80, 3.81)	2.01 (1.57, 2.44)	0.09
placebo vs 5ARIs	3.65 (0.35, 6.98)	0.86 (−0.12, 1.75)	1.05 (0.12, 1.95)	0.11
placebo vs MRAs	0.09 (−2.08, 2.23)	1.61 (−0.11, 3.28)	1.15 (−0.51, 2.81)	0.19
placebo vs PDE5-Is	2.38 (1.77, 2.99)	1.31 (0.20, 2.32)	2.12 (1.55, 2.68)	0.06
placebo vs ABs plus MRAs	1.15 (0.17, 2.14)	3.02 (2.36, 3.76)	2.47 (1.83, 3.18)	0.00
ABs vs 5ARIs	−1.14 (−2.07, −0.33)	0.71 (−1.55, 2.89)	−0.96 (−1.80, −0.14)	0.13
ABs vs PDE5-Is	−0.30 (−1.31, 0.63)	0.37 (−0.38, 1.11)	0.11 (−0.53, 0.75)	0.24
ABs plus 5ARIs vs 5ARIs	−1.29 (−2.43, −0.14)	−2.14 (−5.13, 0.83)	−1.36 (−2.40, −0.35)	0.6
ABs plus MRAs vs ABs	−0.72 (−1.38, −0.12)	1.26 (−1.00, 3.53)	−0.46 (−1.08, 0.08)	0.09
ABs plus PDE5-Is vs ABs	−2.15 (−3.52, −0.77)	−5.08 (−7.07, −3.10)	−3.07 (−4.27, −1.84)	0.02
ABs plus PDE5-Is vs PDE5-Is	−3.55 (−4.99, −2.13)	−1.79 (−3.86, 0.27)	−2.91 (−4.09, −1.69)	0.16
**Qmax**				
placebo vs ABs	−1.03 (−1.37, −0.67)	−1.35 (−2.10, −0.58)	−1.11 (−1.43, −0.79)	0.45
placebo vs 5ARIs	−1.63 (−2.68, −0.59)	−0.99 (−1.81, −0.13)	−1.23 (−1.87, −0.59)	0.34
placebo vs MRAs	0.60 (−1.20, 2.36)	0.01 (−1.48, 1.49)	0.24 (−1.09, 1.56)	0.57
placebo vs PDE5-Is	−0.05 (−0.57, 0.46)	−1.28 (−2.02, −0.49)	−0.40 (−0.94, 0.14)	0.01
placebo vs ABs plus MRAs	−1.16 (−2.38, 0.06)	−0.81 (−1.60, −0.02)	−0.92 (−1.59, −0.18)	0.63
ABs vs 5ARIs	0.15 (−0.62, 0.92)	−0.63 (−1.58, 0.33)	−0.12 (−0.75, 0.49)	0.21
ABs vs PDE5-Is	−0.11 (−0.76, 0.57)	1.24 (0.71, 1.75)	0.71 (0.14, 1.27)	0.00
ABs plus 5ARIs vs 5ARIs	0.66 (−0.36, 1.69)	1.45 (−0.54, 3.46)	0.76 (−0.12, 1.63)	0.48
ABs plus MRAs vs ABs	−0.33 (−1.04, 0.41)	0.77 (−0.88, 2.44)	−0.20 (−0.86, 0.45)	0.24
ABs plus PDE5-Is vs PDE5-Is	1.94 (0.57, 3.33)	0.95 (−0.36, 2.27)	1.50 (0.51, 2.48)	0.28
**IPSS storage subscore**				
placebo vs ABs	0.33 (−0.41, 1.05)	−0.02 (−1.28, 1.29)	0.32 (−0.28, 0.91)	0.62
placebo vs PDE5-Is	0.60 (−0.04, 1.23)	1.09 (−0.13, 2.30)	0.62 (−0.03, 1.26)	0.46
placebo vs ABs plus MRAs	0.79 (−0.76, 2.31)	1.50 (0.54, 2.47)	1.33 (0.50, 2.14)	0.42
ABs vs PDE5-Is	0.51 (−0.55, 1.52)	0.09 (−0.82, 0.99)	0.30 (−0.48, 1.09)	0.53
ABs plus MRAs vs ABs	−1.09 (−1.78, −0.41)	−0.08 (−2.36, 2.14)	−1.00 (−1.66, −0.38)	0.39
ABs plus PDE5-Is vs PDE5-Is	−2.09 (−4.67, 0.30)	−2.11 (−4.76, 0.57)	−1.58 (−3.31, 0.14)	0.99
**IPSS voiding subscore**				
placebo vs ABs	1.16 (0.72, 1.59)	1.18 (0.32, 1.96)	1.17 (0.77, 1.56)	0.98
placebo vs PDE5-Is	1.18 (0.70, 1.63)	0.89 (0.01, 1.74)	1.13 (0.68, 1.55)	0.51
placebo vs ABs plus MRAs	0.39 (−0.53, 1.33)	1.00 (0.37, 1.68)	0.78 (0.23, 1.37)	0.24
ABs vs PDE5-Is	−0.24 (−0.97, 0.50)	0.11 (−0.54, 0.71)	−0.05 (−0.58, 0.46)	0.44
ABs plus MRAs vs ABs	0.31 (−0.24, 0.83)	0.80 (−0.77, 2.36)	0.39 (−0.10, 0.86)	0.54
ABs plus PDE5-Is vs PDE5-Is	−1.66 (−3.55, 0.14)	−2.24 (−4.09, −0.33)	−1.84 (−3.12, −0.56)	0.65

Results of the node split model to assess the inconsistency by testing the difference between the direct effect and indirect effect. If the P-value is more than 0.05, it indicates that the difference between the direct effect and indirect effect was not significant. ABs  =  α-blockers. 5ARIs  =  5α-reductase inhibitors. MRAs  =  muscarinic receptor antagonists. PDE5-Is  =  phosphodiesterase 5 inhibitors.

#### Qmax

**Figure 4:**
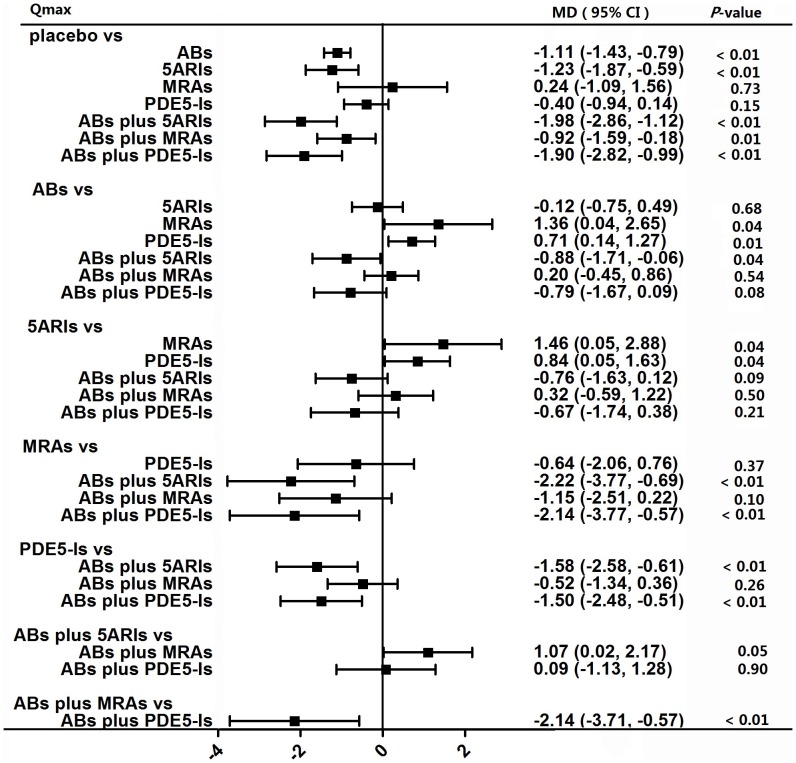
Forest plot for meta-analysis of the overall effect as measured by Qmax. The difference of Qmax between comparisons was calculated as mean difference (MD) and MD above 0 favors the drug therapy on the left header. If the 95% confidence intervals (CI) did not include 0, it means the difference is significant. ABs  =  α-blockers. 5ARIs  =  5α-reductase inhibitors. MRAs  =  muscarinic receptor antagonists. PDE5-Is  =  phosphodiesterase 5 inhibitors.

A total of 55 studies having all seven kinds of oral therapies contributed to the analysis of Qmax. Network meta-analysis (Fig.4) demonstrated that with the exception of monotherapy by MRAs (p = 0.73) and PDE5-Is (p = 0.15), all other five therapies significantly improved Qmax as compared with placebo. Node split models (Table.1) showed that there was significant difference between direct and indirect effect in the comparisons of placebo vs PDE5-Is and ABs vs PDE5-Is. The overall effect in the comparison of ABs vs PDE5-Is was not consistent with the direct effect.

#### IPSS storage subscore

**Figure 5:**
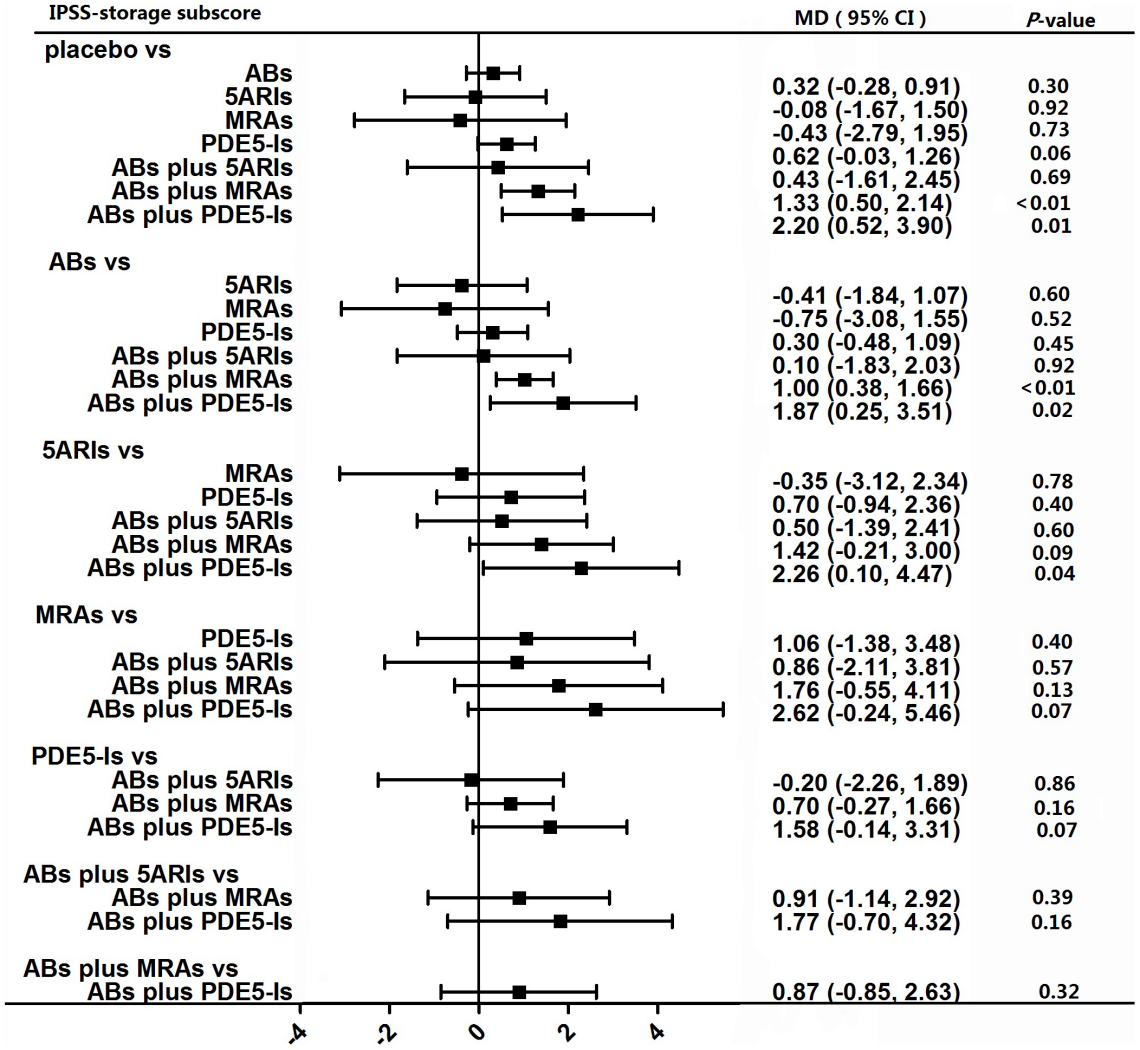
Forest plot for meta-analysis of the overall effect as measured by IPSS storage subscore. The difference of IPSS storage subscore between comparisons was calculated as mean difference (MD) and MD below 0 favors the drug therapy on the left header. If the 95% confidence intervals (CI) did not include 0, it means the difference is significant. ABs  =  α-blockers. 5ARIs  =  5α-reductase inhibitors. MRAs  =  muscarinic receptor antagonists. PDE5-Is  =  phosphodiesterase 5 inhibitors.

A total of 32 studies involving seven kinds of drug therapies contributed to the analysis of IPSS storage subscore. Network meta-analysis (Fig.5) indicated that only the combination therapies of ABs plus PDE5-Is and ABs plus MRAs had a significant effect on the reduction of IPSS storage subscore compared to placebo with a MD of −2.20 (95% CI, −3.90 to −0.52) and −1.33 (95% CI, −2.14 to −0.50), respectively. Node split models (Table.1) did not detect any difference between direct and indirect effect.

#### IPSS voiding subscore

**Figure 6:**
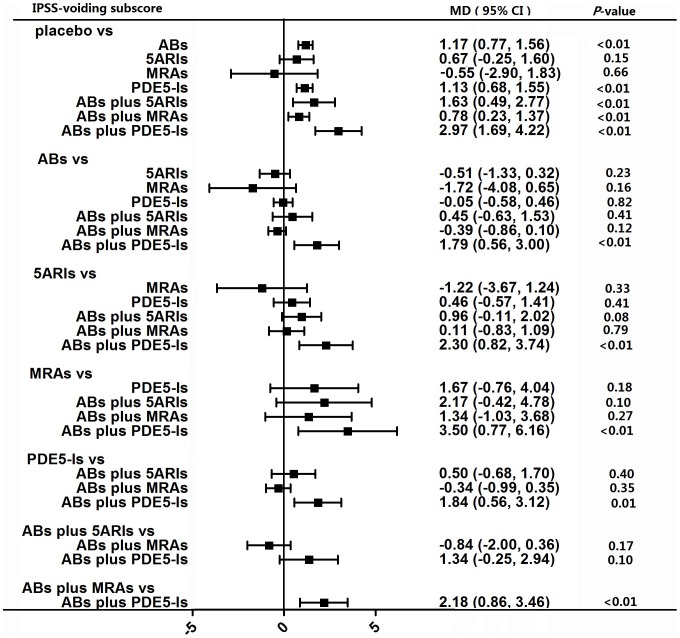
Forest plot for meta-analysis of the overall effect as measured by IPSS voiding subscore. The difference of IPSS voiding subscore between comparisons was calculated as mean difference (MD) and MD below 0 favors the drug therapy on the left header. If the 95% confidence intervals (CI) did not include 0, it means the difference is significant. ABs  =  α-blockers. 5ARIs  =  5α-reductase inhibitors. MRAs  =  muscarinic receptor antagonists. PDE5-Is  =  phosphodiesterase 5 inhibitors.

A total of 29 studies involving all the seven kinds of medical treatments contributed to the analysis of IPSS voiding subscore. As showed in Fig.6, five kinds of medical therapies had a significant effect on the reduction of IPSS voiding subscore as compared with placebo, which were ABs, PDE5-Is, ABs plus 5ARIs, ABs plus MRAs and ABs plus PDE5-Is with a p-value all less than 0.01. Node split models (Table.1) did not detect any difference between direct and indirect effect.

### Rank test

**Figure 7:**
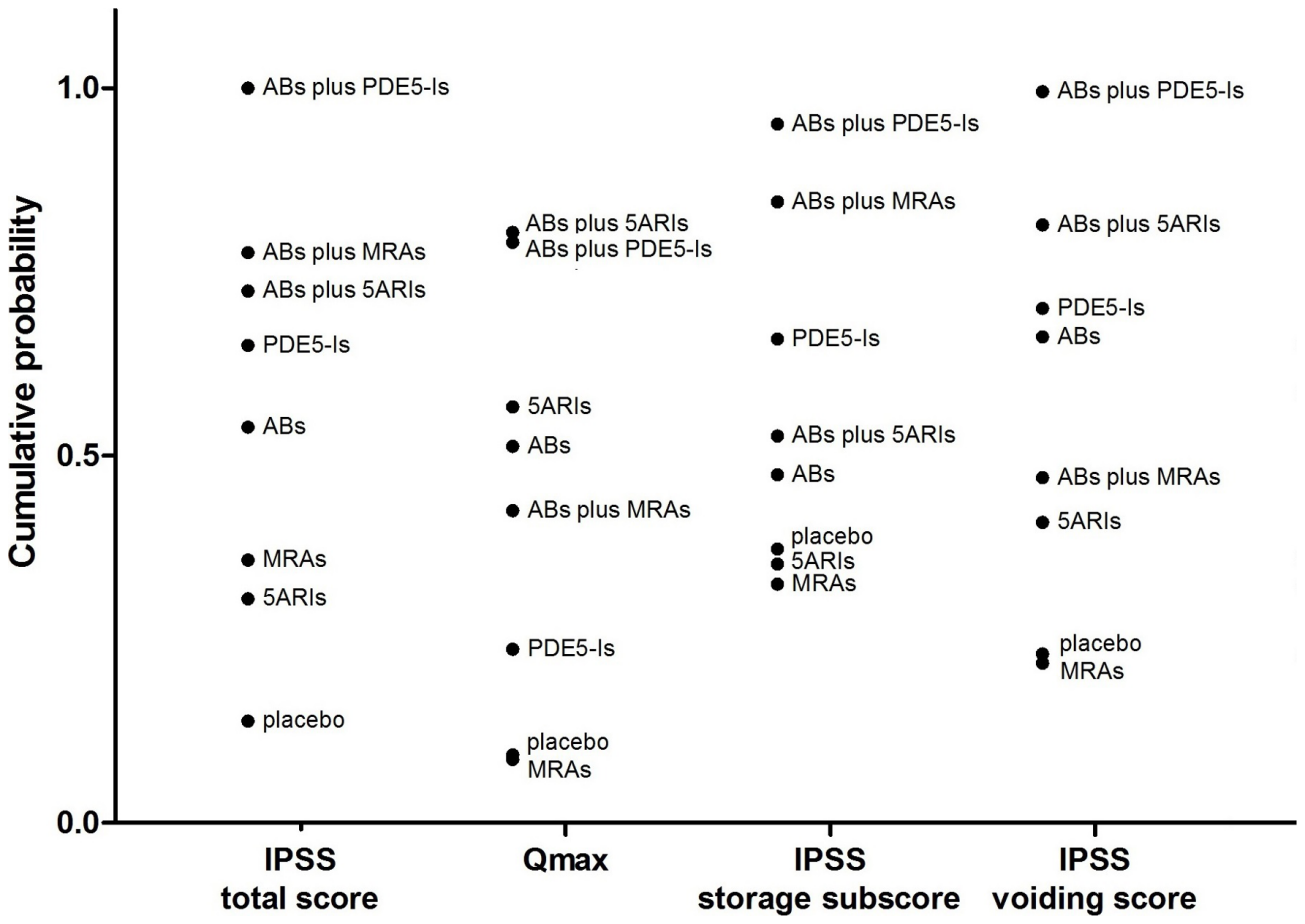
Cumulative probabilities of different kinds of oral drug therapies as measured by the included outcomes. The Bayesian approach could apply the rank probabilities of each drug therapy and the cumulative probability sum the rank probabilities to give an overall probability. Larger cumulative probability represents the better effect on the improvement of IPSS total score, Qmax, IPSS storage subscore and IPSS voiding score, which also represent the rank of the drug therapies. ABs  =  α-blockers. 5ARIs  =  5α-reductase inhibitors. MRAs  =  muscarinic receptor antagonists. PDE5-Is  =  phosphodiesterase 5 inhibitors.

Fig. 7 showed the cumulative probability of all the seven medical therapies and placebo for rank test on each outcome. Among all the drug treatments, combination therapy with ABs plus PDE5-Is ranked highest on the assessment of IPSS total score, storage subscore and voiding subscore. ABs combined with 5ARIs ranked highest for Qmax, but ABs plus 5ARIs and ABs plus PDE5-Is had adjacent cumulative probabilities indicating that these two combination therapies had similar efficacy on improvement of Qmax. Overall, combination therapies resulted in a relatively better effect than monotherapies.

## Discussion

This is the first systematic review and network meta-analysis comparing the effectiveness of different oral drug therapies for LUTS/BPH. Our novel data showed that among all the drug treatments, combination therapy by ABs plus PDE5-Is ranked highest in efficacy for decreasing the IPSS total score, storage subscore and voiding subscore. ABs combined with 5ARIs ranked highest in efficacy for increasing of Qmax. ABs plus MRAs showed great effectiveness on improving storage symptoms, but all monotherapy studies showed no effect on the IPSS storage subscore. PDE5-Is alone also showed promising effect, except on Qmax. The results suggest combination therapies, especially ABs plus PDE5-Is, have greatest efficacy for treatment of LUTS/BPH.

Robert and his colleagues published a systematic review in 2011, in which they retrospectively summarized the recent clinical trials on the assessment of medical treatment for LUTS/BPH [87]. New drugs such as PDE5-Is, and combination therapies such as ABs plus 5ARIs or MRAs were suggested for this disorder. But the selection of therapies should be individualized, based on patients' complaints and the characteristics of different drugs.

In the current review, it was intriguing to find that PDE5-Is combined with ABs ranked highest in patients' subjective symptom evaluations including IPSS total score, IPSS storage subscore and voiding subscore. Although ABs plus 5ARIs ranked highest for Qmax in the rank test, the combination showed adjacent cumulative probabilities as ABs plus PDE5-Is (Fig.7) revealing both these combinations actually shared the highest rank. It is also interesting that PDE5-Is alone demonstrated better efficacy on all the aforementioned outcomes, except Qmax, when compared with other monotherapies including guideline recommended first-line treatment drugs, e.g. ABs and 5ARIs. The use of PDE5-Is (tadalafil 5 mg once daily) for the treatment of BPH/LUTS with or without ED was approved in 2011 in the USA and in 2012 in the European Union. Tadalafil or other PDE5-Is may alleviated LUTS/BPH through several key mechanisms independently [88]–[90]. The effect of PDE5 inhibition leading to increase NO/cGMP concentration in the smooth muscle (SM) of the prostate, urethra, bladder, pelvic neuronal and vascular networks supports lower urinary tract function. Relaxation of the aforementioned SMs results in reduced BPH symptoms including ameliorated detrusor overactivity by increasing blood perfusion and decreasing lower urinary tract tone [91]–[93], rather than simply reducing prostate and urethral compression and obstruction. Moreover, PDE5 inhibition could activate through L-cysteine/hydrogen sulphide pathway in the human bladder, which was newly found and NO-independently [22] [94] and animal studies also indicated that PDE5 inhibition could modulate the activity of afferent-nerve system in the lower urinary tract, relieving the storage symptoms of bladder [89], [95]. Additionally, PDE5-Is were found in vitro to inhibit prostate stromal cell proliferation through attenuating and reverting fibroblast-to-myofibroblast trans-differentiation [96]. In 2012, Gacci et al. conducted an extensive pair-wise meta-analysis on the use of PDE5-Is alone or in combination with ABs for the treatment of LUTS/BPH. They indicated that PDE5-Is could significantly improve LUTS and be a promising treatment for this disorder, although they were ineffective on Qmax. In our network meta-analysis, we confirmed the efficacy of PDE5-Is on LUTS/BPH and we found that the treatment with PDE5-Is did not increase Qmax, either, which was consistent with Gacci's pair-wise meta-analysis. Gacci explained that PDE5-Is concomitant relaxation of the detrusor muscle may counteract the relaxation of the prostate and bladder neck. But for detrusor SM, the role of PDE5-Is may not just be limited to relaxation and the mechanism remains to be fully clarified [97]–[99]. But we did not detect significant difference on the increase of Qmax when comparing ABs plus PDE5-Is with ABs alone, which was inconsistent with Gacci's result. This may be due to the methodology difference that we combined direct and indirect comparisons in our meta-analysis. However, the treatment duration of most trials included in current review was less than 24 weeks, especially for trials with multiple treatment arms. The consistency model of network meta-analysis required rigorous homogeneity between trials but long-term treatment could increase heterogeneity and exaggerate the efficacy of some drugs. Thus we excluded trials with treatment duration more than half a year. Therefore, long-term experience with PDE5-Is in patients with LUTS/BPH is limited. There is also limited information about the reduction of prostate size and no information on slowing of disease progression at present.

In our network meta-analysis of IPSS storage subscore, only the combined therapies of ABs plus PDE5-Is and ABs plus MRAs significantly ameliorated OAB/storage symptoms while all monotherapies showed no effect, including the first-line drugs (MRAs), which have been approved worldwide for treatment of OAB and detrusor overactivity. Consistent with guideline recommendation [9], [10], MRAs should be prescribed with caution when BPH/obstruction exists. However, the current study focused on LUTS/BPH and the effect of treatments on OAB may be underestimated. Although MRAs alone showed no effect on storage symptoms, its combination with ABs efficaciously decreased the storage subscore.

The combined therapy of ABs plus 5ARIs ranked second for IPSS voiding subscore but ranked highest in the test of Qmax. This combination is theoretically ideal with ABs relieving dynamic factor related to prostatic SM tone and 5ARIs attenuating static (anatomical) factors associated with prostatic enlargement. In fact, it is the standard clinical treatment for larger prostate size which involves longer treatment duration [9], [10]. As treatment duration of trials included in the present review was less than 24 weeks, long-term studies are required to definitively determine which combination is better, ABs plus 5ARIs or ABs plus PDE5-Is.

The overall safety profile of oral drug therapies has been confirmed in the included studies. Most cases of adverse events (AEs) were mild to moderate, though some studies reported serious AEs (SAEs), but as shown in Table S1, most SAEs were not considered as treatment-related. Table S3 summarized the common treatment-related AEs. As showed in Table S3, the most commonly reported AEs with ABs were nasopharyngitis, ejaculation disorders and vasodilation effects such as asthenia, headache, dizziness and hypotension, while main AEs of 5ARIs were sexual dysfunction including decreased libido. MRAs could lead to some anticholinergic effects, such as dry mouth and constipation and PDE5-Is could cause flushing, headache, dyspepsia and nasopharyngitis. The overall incidence of AEs for combined therapies was higher than for monotherapies. Moreover, though we did not assess the economic factors in current review, combined therapies could increase medical cost. Therefore, the risk of AEs and medical cost should be consulted with patients when prescribing combined therapies for better effect. In the current, the safety of different class of drugs was not evaluated through network meta-analysis, as the mechanism and type of treatment-related AEs were diverse. Thus it is improper to compare the AEs of different class of drugs.

The overall quality of the included studies was considered acceptable. All included studies had no severe imbalanced baseline, early withdrawal, or other recognizable risk of bias. Moreover, as there was no unified heterogeneity assessment for network meta-analysis, we conducted the node split models to detect the inconsistency between direct and indirect effect and some inconsistency was found, especially in the comparison of ABs vs PDE5-Is on Qmax that the overall effect was not consistent with the direct effect. Thus the conclusion comparing intervention by ABs with PDE5-Is on Qmax should be treated with some caution.

The overall value of the present systematic review and network meta-analysis is lessened by several limitations as follows. Firstly, network meta-analysis was conducted with combining direct and indirect evidences and the overall effect could be influenced by the indirect evidences when the direct comparison was limited. Secondly, as aforementioned, the treatment duration of most trials included in current review was less than 24 weeks and long-term efficacy of different drug therapies for patients with LUTS/BPH was limited. Thirdly, current study aimed on LUTS/BPH and the effect of MRAs for OAB may be underestimated.

## Conclusions

Our novel data demonstrates that ABs plus PDE5-Is was the best combination for treatment for LUTS/BPH in terms of improving outcomes of IPSS total score, IPSS storage subscore and IPSS voiding subscore. ABs plus 5ARIs was the best treatment for increasing Qmax. ABs plus MRAs showed great efficacy on improving storage symptoms. As a newly emerging treatment, there is growing evidence confirming the efficacy of PDE5-Is for the treatment LUTS/BPH, although they consistently exhibited no effect on Qmax. Additionally, all monotherapies including first-line drugs (MRAs) for OAB showed no effect on IPSS storage subscore. Based on our findings, combined therapy, especially ABs plus PDE5-Is, is recommended for short-term treatment for LUTS/BPH. However, further studies are required for longer duration which consider more treatment outcomes, such as disease progression, as well as studies which lead to a greater understanding of the mechanism by which pharmacologic agents, particularly PDE5-Is, are efficacious in treating for BPH/LUTS.

## Supporting Information

Table S1 Characteristics of included studies.
(DOCX)

Table S2 Risk of bias summary. The methodological quality of included studies was appraised with the Cochrane Collaboration bias appraisal tool. In particular, the following factors were evaluated: (1) Adequate sequence generation? (2) Allocation concealment? (3) Binding? (4) Incomplete outcome data addressed? (5) Free of selective reporting? (6) Free of other bias?
(DOCX)

Table S3 Most common reported treatment-related adverse events. We reported the incidence of each common adverse event in the treatment arms and an overall rate summed the incidence.
(DOC)

Checklist S1 (DOC)
