# Supplementary material for: Comparative Effectiveness of Oral Drug Therapies for Lower Urinary Tract Symptoms due to Benign Prostatic Hyperplasia: A Systematic Review and Network Meta-Analysis
Source: PLoS One. 2014 Sep 12;9(9):e107593. doi: 10.1371/journal.pone.0107593 (PMC4162615; doi:10.1371/journal.pone.0107593)
Supplement: Table S1 — Characteristics of included studies. (DOCX) [file pone.0107593.s001.docx]

**Table S1 - Characteristics of included studies**

| Source | Trial design | Sample size | Location | Baseline information | | | | Treatment duration (week) | Trial arms | Outcome | SAEs |
| --- | --- | --- | --- | --- | --- | --- | --- | --- | --- | --- | --- |
|  |  |  |  | Age | IPSS | Qmax | PV |  |  |  |  |
| McVary et al 2007 | RCT DB MC PC | 370 | USA | 60.0 | - | - | - | 12 | sildenafil 50-100mg qd placebo | IPSS total score IPSS storage subscore IPSS voiding subscore Qmax | Y**^1^** |
| McVary et al 2007 | RCT DB MC PC | 281 | USA | 61.5 | 18.0 | 11.4 | - | 12 | tadalafil 5-20mg qd placebo | IPSS total score IPSS storage subscore IPSS voiding subscore Qmax | N |
| Roehrborn et al 2008 | RCT DB MC PC | 1058 | USA | 61.9 | 17.3 | 10.1 | - | 12 | tadalafil 2.5, 5, 10, 20mg qd placebo | IPSS total score IPSS storage subscore IPSS voiding subscore Qmax | Y |
| Stief et al 2008 | RCT DB MC PC | 222 | Germany | 55.9 | 16.8 | 15.9 | - | 8 | vardenafil 10mg bid placebo | IPSS total score IPSS storage subscore IPSS voiding subscore Qmax | Y**^2^** |
| Porst et al 2011 | RCT DB MC PC | 325 | Argentinaet al | 64.8 | 16.8 | 11.7 | - | 12 | tadalafil 5mg qd placebo | IPSS total score IPSS storage subscore IPSS voiding subscore | Y**^3^** |
| Madani et al 2012 | RCT DB PC | 132 | Iran | 64.6 | 13.4 | 9.0 | 41.3 | 12 | tadalafil 10mg qd placebo | IPSS total score Qmax QoL | N |
| Brock et al 2013 | RCT DB MC PC | 1089 | Argentinaet al | 63.3 | 19.5 | - | - | 12 | tadalafil 5mg qd placebo | IPSS total score IPSS storage subscore IPSS voiding subscore | N |
| Roehrborn et al 2010 | RCT DB MC PC | 1058 | USA | - | - | - | - | 12 | tadalafil 2.5, 5, 10, 20mg qd placebo | Qmax | N |
| Dmochowski et al 2010 | RCT DB MC PC | 200 | USA Canada | 58.6 | 21.8 | 14.3 | - | 12 | tadalafil 20mg qd placebo | IPSS total score IPSS storage subscore IPSS voiding subscore Qmax | N |
| Kaplan et al 2013 | RCT DB MC PC | 222 | Europe USA | 64.6 | 17.8 | 7.6 | - | 12 | tamsulosin 0.4mg qd +solifenacin 6mg qd tamsulosin 0.4mg qd +solifenacin 9mg qd placebo | IPSS total score IPSS storage subscore IPSS voiding subscore Qmax | N |
| Nording 2005 | RCT DB MC PC | 625 | Europe Israel | 64.5 | 20.0 | 8.9 | - | 12 | alfuzosin 10, 15mg qd tamsulosin 0.4mg qd  placebo | IPSS total score IPSS storage subscore IPSS voiding subscore | Y |
| Roehrborn 2001 | RCT DB MC PC | 536 | USA Canada | 63.6 | 21.4 | 8.7 | 38.4 | 12 | alfuzosin 10, 15mg qd placebo | IPSS total score IPSS storage subscore IPSS voiding subscore Qmax | N |
| Kirby et al 2001 | RCT DB MC PC | 1475 | Europe | 64.0 | 18.1 | 10.3 | - | 13 | doxazosin 4mg-8mg qd doxazosin 1mg-8mg qd placebo | IPSS total score Qmax | N |
| Lloyd et al 1992 | RCT DB MC PC | 93 | UK | 65.7 | - | 8.0 | - | 12 | prazosin 0.5-2mg bid placebo | Qmax | N |
| Elhilali et al 1996 | RCT DB MC PC | 164 | Canada | 64.5 | - | 10.2 | - | 24 | terazosin 1mg-10mg qd placebo | Qmax | N |
| Kirby et al 1998 | RCT DB MC PC | 353 | UK | 65.3 | 18.5 | 10.1 | 30.0 | 12 | doxazosin 1-2mg morning or evening dosing qd placebo | IPSS total score Qmax | N |
| Ozbey et al 1999 | RCT PC | 57 | Turkey | - | - | 10.4 | - | 24 | doxazosin 2-4mg qd placebo | Qmax | N |
| Van et al 2000 | RCT DB MC PC | 447 | France et al | 64.6 | 17.3 | 9.1 | - | 12 | alfuzosin 10mg qd alfuzosin 2.5mg tid placebo | IPSS total score IPSS storage subscore IPSS voiding subscore Qmax | Y |
| Jardin et al 1991 | RCT DB MC PC | 518 | Europe | 65.4 | - | 12.0 | - | 13 | alfuzosin 7.5-10mg qd placebo | Qmax | N |
| Roehrborn ＆ Siegel 1996 | RCT DB MC PC | 327 | Europe | 63.4 | - | 10 | - | 16 | doxazosin 0.5-12mg qd placebo | Qmax | N |
| Andersen et al 2000 | RCT DB MC PC | 795 | Denmark et al | 65.2 | 17.8 | 10.1 | - | 17 | doxazosin 4mg-8mg qd doxazosin 1mg-8mg qd placebo | IPSS total score Qmax | N |
| Chapple et al 1994 | RCT DB PC | 135 | UK | - | - | 9.1 | - | 14 | doxazosin 1-4mg qd placebo | Qmax | N |
| Brawer et al 1993 | RCT DB MC PC | 160 | USA | 64 | - | 8.7 | - | 24 | terazosin 1-10mg qd placebo | Qmax | N |
| Fabricius et al 1990 | RCT DB MC PC | 296 | Europe | 63.7 | - | 10.6 | - | 14 | tamsulosin 0.4mg qd placebo | IPSS total score IPSS storage subscore IPSS voiding subscore Qmax | N |
| Abrams et al 1997 | RCT DB MC PC | 126 | UK | 65.0 | - | 9.8 | 32.5 | 4 | tamsulosin 0.2, 0.4, 0.6, qd placebo | Qmax | Y**^4^** |
| Chapple et al 1996 | RCT DB MC PC | 575 | Europe | 64.0 | - | 10.1 | - | 12 | tamsulosin 0.4mg qd placebo | Qmax | N |
| Lepor 1998 | RCT DB MC PC | 756 | USA | - | 19.8 | 9.6 | - | 13 | tamsulosin 0.4, 0.8mg qd placebo | IPSS total score Qmax | Y |
| Narayan ＆ Tewari 1998 | RCT DB MC PC | 735 | USA | 58 | - | - | - | 13 | tamsulosin 0.4, 0.8mg qd placebo | IPSS total score Qmax | Y**^5^** |
| Christensen et al 1993 | RCT DB MC PC | 100 | Denmark et al | 67.5 | - | 7.6 | - | 9 | doxazosin 4mg qd placebo | Qmax | N |
| Jin et al 2011 | RCT MC | 250 | China | 61.4 | 17.9 | 10.9 | - | 12 | doxazosin 4mg qd +sildenafil 25-100mg on demand sildenafil 25-100mg on demand | IPSS total score IPSS storage subscore IPSS voiding subscore Qmax | N |
| Arora et al 2012 | RCT | 40 | India | 60.0 | 15.6 | 9.4 | 53.4 | 24 | tamsulosin 0.4mg qd +dutasteride 0.5mg qd tamsulosin 0.4mg qd | IPSS total score Qmax | N |
| Ozturk et al 2012 | RCT | 100 | Turkey | 60.2 | 19.9 | 10.4 | 46.2 | 12 | alfuzosin 10mg qd +sildenafil 50mg qd alfuzosin 10mg qd | IPSS total score Qmax | N |
| Regadas et al 2013 | RCT DB | 40 | Brazil | 60.4 | 20.5 | 6.8 | 43.4 | 4 | tamsulosin 0.4mg +tadalafil 5mg qd tamsulosin 0.4mg qd | IPSS total score IPSS storage subscore IPSS voiding subscore Qmax | N |
| Gacci et al 2012 | RCT DB | 60 | Italy | 68.0 | 16.5 | 10.7 | - | 12 | tamsulosin 0.4mg +vardenafil 10mg tamsulosin 0.4mg | IPSS total score Qmax | N |
| Lee et al 2005 | RCT DB MC | 211 | Korea | 66.0 | 21.5 | 10.4 | - | 8 | propiverine 20mg qd +doxazosin 4mg qd doxazosin 4mg qd | IPSS total score IPSS storage subscore IPSS voiding subscore Qmax | N |
| MacDiarmid et al 2008 | RCT DB MC | 420 | USA | 62.9 | 20.4 | 15.1 | - | 12 | tamsulosin 0.4mg +oxybutynin 10mg qd tamsulosin 0.4mg qd | IPSS total score IPSS storage subscore Qmax | Y**^6^** |
| Yamaguchi et al 2011 | RCT DB MC | 769 | Japan | 70.0 | 13.9 | 13.6 | 34.1 | 12 | tamsulosin 0.2mg qd +solifenacin 2.5mg qd tamsulosin 0.2mg qd +solifenacin 5mg qd tamsulosin 0.2mg qd | IPSS total score IPSS storage subscore IPSS voiding subscore Qmax | N |
| Maruyama et al 2006 | RCT DB | 101 | Japan | 67.3 | 17.1 | 11.1 | 32.5 | 12 | naftopidil 25-75mg qd +propiverine 10-20mg qd  naftopidil 25-75mg qd +oxybutynin 2-6mg qd naftopidil 25-75mg qd | IPSS total score Qmax | N |
| Yang et al 2007 | RCT | 191 | China | 69.1 | 20.0 | - | 39.5 | 6 | terazosin 2mg qd +tolterodine 2mg bid terazosin 2mg qd | IPSS total score IPSS storage subscore IPSS voiding subscore Qmax | N |
| Shen et al 2011 | RCT | 74 | China | 73.9 | 21.2 | 14.0 | -47+49 | 12 | terazosin 2mg qd +tolterodine 2mg bid terazosin 2mg qd | IPSS total score IPSS storage subscore Qmax | N |
| Nishizawa et al 2011 | RCT MC | 214 | Japan | 70.2 | 13.0 | 12.0 | - | 12 | tamsulosin 0.2mg qd +propiverine 10mg qd tamsulosin 0.2mg qd +propiverine 20mg qd tamsulosin 0.2mg qd | IPSS storage subscore | N |
| Seo et al 2011 | RCT | 57 | Korea | 57.8 | 17.8 | 14.6 | 22.7 | 12 | tamsulosin 0.2mg qd +solifenacin 5mg qd tamsulosin 0.2mg qd | IPSS total score IPSS storage subscore IPSS voiding subscore Qmax | N |
| Lee et al 2011 | RCT DB MC | 176 | Korea | 61.2 | 21.4 | 10.9 | 34.5 | 12 | doxazosin 4mg qd +tolterodine 4mg qd doxazosin 4mg qd | IPSS total score IPSS storage subscore IPSS voiding subscore | N |
| Bae et al 2011 | RCT MC | 209 | Korea | 63.2 | 21.7 | 14.8 | 26.4 | 8 | alfuzosin10mg qd +propiverine 10mg qd alfuzosin 10mg qd | IPSS total score IPSS storage subscore IPSS voiding subscore Qmax | N |
| Kirby et al 1992 | RCT DB PC | 69 | UK | - | - | 8.8 | 50.0 | 12 | finasteride 5, 10mg qd placebo | Qmax | N |
| Stoner 1992 | RCT DB MC PC | 190 | USA | 64.4 | - | - | - | 12 | finasteride 0.2-40mg qd finasteride 5-80mg qd placebo | Qmax | N |
| Yu et al 1995 | RCT DB MC PC | 46 | Taiwan | 65.8 | 18.1 | 11.3 | 23.6 | 24 | finasteride 5mg qd placebo | IPSS total score Qmax | N |
| Na et al 2012 | RCT DB MC PC | 253 | China | 66.4 | 18.3 | 11.7 | 45.2 | 12 | dutasteride 0.5mg qd placebo | Qmax | Y**^7^** |
| Tammela ＆ Kontturi 1993 | RCT DB PC | 36 | Finland | 65.0 | - | 8.3 | 49.0 | 24 | finasteride 5mg qd placebo | Qmax | N |
| Rigatti et al 2003 | RCT DB MC | 441 | Italy | 63.0 | 16.6 | 10.8 | 39 | 24 | tamsulosin 0.4mg qd finasteride 5mg qd | IPSS total score IPSS storage subscore IPSS voiding subscore Qmax | Y |
| Lee 2002 | RCT | 205 | Korea | 64.7 | 19.5 | 9.4 | 29.8 | 24 | tamsulosin 0.2mg qd finasteride 5mg qd | IPSS total score Qmax | N |
| Singh et al 2013 | RCT | 69 | India | 67.8 | 20.1 | - | 38.1 | 12 | tamsulosin 0.4mg qd finasteride 5mg qd | IPSS total score | N |
| Yokoyama et al 2013 | RCT DB MC PC | 784 | Japan et al | 63.1 | 16.8 | 12.4 | - | 12 | tadalafil 2.5, 5mg qd tamsulosin 0.2mg qd  placebo | IPSS total score IPSS storage subscore IPSS voiding subscore Qmax | Y**^8^** |
| Kim et al 2011 | RCT DB MC PC | 151 | Korea | 61.6 | 17.4 | 11.2 | - | 12 | tadalafil 5mg qd tamsulosin 0.2mg qd placebo | IPSS total score IPSS storage subscore IPSS voiding subscore Qmax | N |
| Oelke et al 2012 | RCT DB MC PC | 511 | Australia et al | 63.6 | 17.2 | 9.9 | - | 12 | tadalafil 5mg qd tamsulosin 0.4mg qd placebo | IPSS total score IPSS storage subscore IPSS voiding subscore Qmax | Y |
| Van et al 2013 | RCT DB MC PC | 1334 | Europe, Australia | 65.4 | 18.7 | 8.9 | 38.1 | 12 | tamsulosin 0.4mg qd +solifenacin 6mg qd tamsulosin 0.4mg qd +solifenacin 9mg qd  tamsulosin 0.4mg qd  placebo | IPSS total score IPSS storage subscore IPSS voiding subscore | Y**^9^** |
| Liguori et al 2009 | RCT MC | 66 | Italy | 61.3 | 15 | 12.3 | - | 12 | alfuzosin 10mg qd + tadalafil 20mg every other day alfuzosin 10mg qd  tadalafil 20mg every other day | IPSS total score IPSS storage subscore IPSS voiding subscore Qmax | N |
| Kaplan et al 2007 | RCT DB | 62 | USA | 63.4 | 17.4 | 9.5 | - | 12 | alfuzosin 10mg qd +sildenafil 25mg qd alfuzosin 10mg qd sildenafil 25mg qd | IPSS total score Qmax | N |
| Tuncel et al 2010 | RCT | 60 | Turkey | 58 | 15.3 | 14.2 | - | 8 | tamsulosin 0.4mg qd + sildenafil 25mg 4days/week tamsulosin 0.4mg qd sildenafil 25mg 4days/week | Qmax | N |
| Yokoyama et al 2009 | RCT | 66 | Japan | 69.1 | 18.1 | 9.8 | 26.6 | 4 | naftopidil 50mg qd +propiverine 20mg qd naftopidil 50mg qd propiverine 20mg qd | IPSS total score IPSS storage subscore IPSS voiding subscore Qmax | N |
| COMBAT**^10^** | RCT DB MC | 4844 | USA et al | 66.1 | 16.4 | 10.7 | 55 | 24 | dutasteride 0.5mg qd +tamsulosin 0.4mg qd dutasteride 0.5mg qd tamsulosin 0.4mg qd | IPSS total score IPSS storage subscore IPSS voiding subscore Qmax | Y**^11^** |
| Debruyne et al 1998 | RCT DB MC | 1051 | France et al | 63.3 | 15.4 | 9.9 | 41.2 | 24 | alfuzosin 5mg bid +finasteride 5mg qd alfuzosin 5mg bid finasteride 5mg qd | IPSS total score Qmax | N |
| Roehrborn et al 2009 | RCT DB MC PC | 879 | USA | 61.9 | 19.9 | 12.9 | 35.0 | 12 | tamsulosin 0.4mg +tolterodine 4mg tolterodine 4mg tamsulosin 0.4mg placebo | Qmax | N |
| Van et al 2013 | RCT DB MC PC | 937 | Europe | 65.2 | 18.3 | 10.2 | 39.5 | 12 | solifenacin 3, 6, 9mg qd +tamsulosin 0.4mg qd tamsulosin 0.4mg qd solifenacin 3, 6, 9mg qd placebo | IPSS total score | Y |
| Kawabe et al 2006 | RCT DB MC PC | 265 | Japan | 65.5 | 17.1 | 9.9 | 35.6 | 12 | silodosin 4mg bid  placebo | IPSS total score IPSS storage subscore IPSS voiding subscore Qmax | N |
| Marks et al 2009 | RCT DB MC PC | 923 | USA | 64.6 | 21.3 | 8.8 | - | 12 | Silodosin 8mg qd  placebo | IPSS total score IPSS storage subscore IPSS voiding subscore Qmax | N |

**Notes: ^1^** A 71-year old man had a severe acute cerebrovascular stroke after receiving 100mg sildenafil for 32 days. **^2^** None of the serious AEs were considered treatment related. **^3^** An 81 yr old man with several cardiovascular risk factors died from an acute myocardial infarction, which may be assessed as possiblely related to study drug. **^4^** Epistaxia in placebo group and arthralgia in tamsulosin group resulted in discontinuation from the study. Chest pain in tamsulosin group was considered unrelated to study drug. **^5^** SAEs reported were chest pain, acute myocardial infarction, neutropenia, abdominal aortic aneurysm and diverticulosis. The authors did not assess whether these SAEs were related to study drug. **^6^** None of the SAEs were considered treatment related. **^7^** One in the dutasteride group experienced glaucoma, but none of the serious AEs were considered treatment related. **^8^** One patient with injury was unable to be contacted and none of the other SAEs were considered treatment related. **^9^** Two patients died from myocardial infarction and small cell lung cancer but neither death was considered related to study treatment. Drug related SAEs were urinary retention and atrial fibrillation.**^10^** The Combination of Avodart and Tamsulosin (CombAT) study was an ongoing, international, double-blind, randomized, parallel-group study and we extracted the data of outcome at 6-month from the beginning of treatment. **^11^** All the drug related SAEs were less than 1% in each group.

**Abbreviations:** RCT, randomized controlled trial; MC, multiple centers; DB, double blind; PC, placebo controlled; AEs, adverse events; SAEs, serious adverse events; IPSS, International Prostate Symptom Score; IIEF, International Index of Erectile Function; Qmax, maximum flow rate; PVR, post voided residue; USA, United States of America; UK, United Kingdom.
