# Supplementary material for: Comparative Effectiveness of Oral Drug Therapies for Lower Urinary Tract Symptoms due to Benign Prostatic Hyperplasia: A Systematic Review and Network Meta-Analysis
Source: PLoS One. 2014 Sep 12;9(9):e107593. doi: 10.1371/journal.pone.0107593 (PMC4162615; doi:10.1371/journal.pone.0107593)
Supplement: Table S2 — Risk of bias summary. The methodological quality of included studies was appraised with the Cochrane Collaboration bias appraisal tool. In particular, the following factors were evaluated: (1) Adequate sequence generation? (2) Allocation concealment? (3) Binding? (4) Incomplete outcome data addressed? (5) Free of selective reporting? (6) Free of other bias? (DOCX) [file pone.0107593.s002.docx]

**Table S2 - Risk of bias summary**

| Source | Adequate sequence generation? | Allocation concealment? | Blinding? | Incomplete outcome data addressed? | Free of selective reporting? | Free of other bias? |
| --- | --- | --- | --- | --- | --- | --- |
| McVary et al 2007 | Y | U | Y | Y | Y | Y |
| McVary et al 2007 | U | U | Y | Y | Y | Y |
| Roehrborn et al 2008 | U | U | Y | Y | Y | Y |
| Stief et al 2008 | Y | U | Y | Y | Y | Y |
| Porst et al 2011 | N**^1^** | U | Y | Y | Y | Y |
| Madani et al 2012 | Y | U | Y | U | Y | Y |
| Brock et al 2013 | U | U | Y | U | Y | Y |
| Roehrborn et al 2010 | U | U | Y | U | Y | Y |
| Dmochowski et al 2010 | U | U | Y | Y | Y | Y |
| Kaplan et al 2013 | U | U | Y | Y | Y | Y |
| Nording 2005 | U | U | Y | Y | Y | Y |
| Roehrborn 2001 | U | U | Y | Y | Y | Y |
| Kirby et al 2001 | U | U | Y | Y | Y | Y |
| Lloyd et al 1992 | U | U | Y | Y | Y | Y |
| Elhilali et al 1996 | U | U | Y | Y | Y | Y |
| Kirby et al 1998 | U | U | Y | U | Y | Y |
| Ozbey et al 1999 | U | U | U | N**^2^** | Y | Y |
| Van et al 2000 | U | U | Y | Y | Y | Y |
| Jardin et al 1991 | U | U | Y | Y | Y | Y |
| Roehrborn ＆ Siegel 1996 | U | U | Y | Y | Y | Y |
| Andersen et al 2000 | U | U | Y | U | Y | Y |
| Chapple et al 1994 | U | U | Y | Y | Y | Y |
| Brawer et al 1993 | Y | Y | Y | U | Y | Y |
| Fabricius et al 1990 | U | Y | Y | Y | Y | Y |
| Abrams et al 1997 | U | Y | Y | U | Y | Y |
| Chapple et al 1996 | U | U | Y | Y | Y | Y |
| Lepor et al 1998 | U | U | Y | U | Y | Y |
| Narayan ＆ Tewari 1998 | U | U | Y | Y | Y | Y |
| Christensen et al 1993 | Y | U | Y | U | Y | Y |
| Jin et al 2011 | U | U | Y | U | Y | Y |
| Arora et al 2012 | U | U | U | U | Y | Y |
| Ozturk et al 2012 | U | U | Y | Y | Y | Y |
| Regadas et al 2013 | N**^3^** | U | Y | U | Y | Y |
| Gacci et al 2012 | Y | U | Y | Y | Y | Y |
| Lee et al 2005 | Y | U | Y | Y | Y | Y |
| MacDiarmid et al 2008 | U | U | Y | U | Y | Y |
| Yamaguchi et al 2011 | U | U | Y | Y | Y | Y |
| Maruyama et al 2006 | Y | U | Y | Y | Y | Y |
| Yang et al 2007 | Y | U | Y | U | Y | Y |
| Shen et al 2011 | U | U | U | U | Y | Y |
| Nishizawa 2011 | U | U | U | N**^4^** | Y | Y |
| Seo et al 2011 | Y | U | Y | U | Y | Y |
| Lee et al 2011 | Y | Y | Y | N**^5^** | Y | Y |
| Bae et al 2011 | Y | U | Y | U | Y | Y |
| Kirby et al 1992 | U | U | Y | U | Y | Y |
| Stoner 1992 | U | U | Y | U | Y | Y |
| Yu et al 1995 | U | U | Y | Y | Y | Y |
| Na et al 2012 | Y | U | Y | Y | Y | Y |
| Tammela ＆ Kontturi 1993 | U | U | Y | Y | Y | Y |
| Rigatti et al 2003 | U | Y | Y | Y | Y | Y |
| Lee et al 2002 | U | U | Y | Y | Y | Y |
| Singh et al 2013 | U | U | U | Y | Y | Y |
| Yokoyama et al 2013 | Y | U | Y | Y | Y | Y |
| Kim et al 2011 | N**^6^** | U | Y | Y | Y | Y |
| Oelke et al 2012 | N^7^ | U | Y | Y | Y | Y |
| Van et al 2013 | Y | U | Y | Y | Y | Y |
| Liguori et al 2009 | U | U | Y | U | Y | Y |
| Kaplan et al 2007 | U | U | Y | U | Y | Y |
| Tuncel et al 2010 | U | U | Y | U | Y | Y |
| Yokoyama et al 2009 | N**^8^** | U | Y | U | Y | Y |
| COMBAT 2008 | Y | Y | Y | Y | Y | Y |
| Debruyne et al 1998 | U | U | Y | Y | Y | Y |
| Roehrborn et al 2009 | U | U | Y | U | Y | Y |
| Van et al 2013 | U | U | Y | Y | Y | Y |
| Kawabe et al 2006 | U | U | Y | Y | Y | Y |
| Marks et al 2009 | Y | Y | Y | Y | Y | Y |

**Notes: ^1^** Randomization was stratified by baseline LUTS severity, geographic region and history of ED. **^2^** 10 of 28 in placebo and 6 of 29 in doxazosin group did no complete the invasive urodynamic assessment, but no explanation or more information reported. **^3^** Randomization was stratified by baseline obstructive index and baseline LUTS severity. **^4^** 73 of 214 participants withdrawed with no detailed reasons for withdraw reported. **^5^** 49 of 176 participants lost in the follow-up with no detailed reasons reported. **^6^** Randomization was stratified by prior alpha-blocker use and LUTS severity at baseline. **^7^** Randomization was stratified by baseline LUTS severity, geographic region, and patient query regarding prior ED diagnosis. **^8^** Participants were divided according to daily urinary urgency episodes. Y= yes. N = no. U = unclear.
