# Supplementary material for: Comparative Effectiveness of Oral Drug Therapies for Lower Urinary Tract Symptoms due to Benign Prostatic Hyperplasia: A Systematic Review and Network Meta-Analysis
Source: PLoS One. 2014 Sep 12;9(9):e107593. doi: 10.1371/journal.pone.0107593 (PMC4162615; doi:10.1371/journal.pone.0107593)
Supplement: Table S3 — Most common reported treatment-related adverse events. We reported the incidence of each common adverse event in the treatment arms and an overall rate summed the incidence. (DOC) [file pone.0107593.s003.doc]

**Table S3 – Most common reported treatment-related adverse events**

| Common AEs | ABs | 5ARI | MRAs | PDE5-Is | ABs plus 5ARIs | ABs plus MRAs | ABs plus PDE5-Is | Source |
| --- | --- | --- | --- | --- | --- | --- | --- | --- |
| asthenia | 20/470 |  |  |  |  |  |  | Nording 2005 |
|  | 7/335 |  |  |  |  |  |  | Roehrborn 2001 |
|  | 71/1291 |  |  |  |  |  |  | Kirby et al 2001 |
|  | 2/326 |  |  |  |  | 5/661 |  | Van et al 2013 |
|  | 2/177 |  | 0/129 |  |  | 1/532 |  | Van et al 2013 |
|  | 10/81 |  |  |  |  |  |  | Elhilali et al 1996 |
|  | 18/234 |  |  |  |  |  |  | Kirby et al 1998 |
|  | 6/293 |  |  |  |  |  |  | Van et al 2000 |
|  | 5/251 |  |  |  |  |  |  | Jardin et al 1991 |
|  | 21/196 |  |  |  |  |  |  | Roehrborn＆Siegel 1996 |
|  | 26/629 |  |  |  |  |  |  | Andersen et al 2000 |
|  | 6/73 |  |  |  |  |  |  | Brawer et al 1993 |
|  | 4/97 |  |  |  |  |  |  | Abrams et al 1997 |
|  | 25/501 |  |  |  |  |  |  | Lepor 1998 |
|  | 56/492 |  |  |  |  |  |  | Naraya＆Tewari 1998 |
|  | ***5.12%*** |  | ***0*** |  |  | ***0.5%*** |  | ***overall*** |
| headache |  |  |  | 5/82 |  |  | 9/168 | Jin et al 2011 |
|  |  |  |  |  |  | 8/148 |  | Kaplan et al 2013 |
|  | 7/168 |  |  | 5/171 |  |  |  | Oelke et al 2012 |
|  | 1/103 | 0/102 |  |  |  |  |  | Lee 2002 |
|  | 1/152 |  |  | 6/306 |  |  |  | Yokoyama et al 2013 |
|  | 1/20 |  |  |  | 0/20 |  |  | Arora et al 2012 |
|  | 1/46 | 0/14 |  |  |  |  |  | Singh et al 2013 |
|  | 0/49 |  |  | 1/51 |  |  |  | Kim et al 2011 |
|  |  |  |  | 21/189 |  |  |  | McVary et al 2007 |
|  |  |  |  | 3/138 |  |  |  | McVary et al 2007 |
|  |  |  |  | 29/845 |  |  |  | Roehrborn et al 2008 |
|  |  |  |  | 14/108 |  |  |  | Stief et al 2008 |
|  |  |  |  | 6/161 |  |  |  | Porst et al 2011 |
|  |  |  |  | 17/544 |  |  |  | Brock et al 2013 |
|  |  |  |  | 7/99 |  |  |  | Dmochowski et al 2010 |
|  | 13/335 |  |  |  |  |  |  | Roehrborn 2001 |
|  | 73/1291 |  |  |  |  |  |  | Kirby et al 2001 |
|  | 2/326 |  |  |  |  | 4/661 |  | Van et al 2013 |
|  | 2/177 |  | 0/179 |  |  | 4/532 |  | Van et al 2013 |
|  | 6/81 |  |  |  |  |  |  | Elhilali et al 1996 |
|  | 39/234 |  |  |  |  |  |  | Kirby et al 1998 |
|  | 5/293 |  |  |  |  |  |  | Van et al 2000 |
|  | 16/251 |  |  |  |  |  |  | Jardin et al 1991 |
|  | 21/196 |  |  |  |  |  |  | Roehrborn ＆ Siegel 1996 |
|  | 31/639 |  |  |  |  |  |  | Andersen et al 2000 |
|  | 5/73 |  |  |  |  |  |  | Brawer et al 1993 |
|  | 6/197 |  |  |  |  |  |  | Fabricius et al 1990 |
|  | 2/97 |  |  |  |  |  |  | Abrams et al 1997 |
|  | 93/501 |  |  |  |  |  |  | Lepor 1998 |
|  | 108/492 |  |  |  |  |  |  | Narayan ＆ Tewari 1998 |
|  | ***7.57%*** | ***0*** | ***0*** | ***4.23%*** | ***0*** | ***1.19%*** | ***5.36%*** | ***overall*** |
| dizziness |  |  |  | 4/82 |  |  | 8/168 | Jin et al 2011 |
|  | 1/77 |  |  |  |  | 1/132 |  | Bae et al 2011 |
|  | 6/358 | 4/344 |  |  | 8/349 |  |  | Debruyne et al 1998 |
|  | 6/168 |  |  | 4/171 |  |  |  | Oelke et al 2012 |
|  | 1/20 |  |  | 0/21 |  |  | 1/21 | Kaplan et al 2007 |
|  | 0/69 |  |  |  |  | 8/142 |  | Lee et al 2005 |
|  | 2/152 |  |  | 3/306 |  |  |  | Yokoyama et al 2013 |
|  | 1/44 |  |  |  |  | 0/36 |  | Maruyama et al 2006 |
|  | 1/20 |  |  |  | 0/20 |  |  | Arora et al 2012 |
|  | 2/46 | 1/14 |  |  |  |  |  | Singh et al 2013 |
|  | 0/36 |  |  |  | 1/33 |  |  | Yang et al 2007 |
|  | 0/60 |  |  |  |  | 1/122 |  | Nishizawa et al 2011 |
|  |  |  |  | 8/544 |  |  |  | Brock et al 2013 |
|  | 23/470 |  |  |  |  |  |  | Nording 2005 |
|  | 29/335 |  |  |  |  |  |  | Roehrborn 2001 |
|  | 94/1291 |  |  |  |  |  |  | Kirby et al 2001 |
|  | 3/177 |  | 1/179 |  |  | 4/532 |  | Van et al 2013 |
|  | 27/1611 | 11/1623 |  |  | 26/1610 |  |  | COMBAT |
|  | 9/81 |  |  |  |  |  |  | Elhilali et al 1996 |
|  | 33/234 |  |  |  |  |  |  | Kirby et al 1998 |
|  | 10/293 |  |  |  |  |  |  | Van et al 2000 |
|  | 18/251 |  |  |  |  |  |  | Jardin et al 1991 |
|  | 34/196 |  |  |  |  |  |  | Roehrborn ＆ Siegel 1996 |
|  | 45/639 |  |  |  |  |  |  | Andersen et al 2000 |
|  | 15/73 |  |  |  |  |  |  | Brawer et al 1993 |
|  | 5/198 |  |  |  |  |  |  | Fabricius et al 1990 |
|  | 4/97 |  |  |  |  |  |  | Abrams et al 1997 |
|  | 53/501 |  |  |  |  |  |  | Lepor 1998 |
|  | 106/492 |  |  |  |  |  |  | Narayan ＆ Tewari 1998 |
|  | ***6.61%*** | ***0.81%*** | ***0.56%*** | ***1.69%*** | ***1.74%*** | ***1.45%*** | ***4.76%*** | ***overall*** |
| flushing |  |  |  | 4/82 |  |  | 6/168 | Jin et al 2011 |
|  | 0/20 |  |  | 1/21 |  |  | 0/21 | Kaplan et al 2007 |
|  | 0/49 |  |  | 1/51 |  |  |  | Kim et al 2011 |
|  |  |  |  | 9/189 |  |  |  | McVary et al 2007 |
|  | ***0*** |  |  | ***4.37%*** |  |  | ***3.17%*** | ***overall*** |
| dyspepsia |  |  |  | 2/82 |  |  | 5/168 | Jin et al 2011 |
|  | 3/168 |  |  | 4/171 |  |  |  | Oelke et al 2012 |
|  | 1/103 | 0/102 |  |  |  |  |  | Lee 2002 |
|  | 0/20 |  |  | 1/21 |  |  | 0/21 | Kaplan et al 2007 |
|  | 1/69 |  |  |  |  | 5/142 |  | Lee et al 2005 |
|  | 1/36 |  |  |  |  | 0/33 |  | Yang et al 2007 |
|  | 1/49 |  |  | 0/51 |  |  |  | Kim et al 2011 |
|  |  |  |  | 12/189 |  |  |  | McVary et al 2007 |
|  |  |  |  | 5/138 |  |  |  | McVary et al 2007 |
|  |  |  |  | 28/845 |  |  |  | Roehrborn et al 2008 |
|  |  |  |  | 8/108 |  |  |  | Stief et al 2008 |
|  |  |  |  | 15/544 |  |  |  | Brock et al 2013 |
|  |  |  |  | 8/99 |  |  |  | Dmochowski et al 2010 |
|  | 3/355 |  |  |  |  |  |  | Roehrborn 2001 |
|  | 1/326 |  |  |  |  | 10/661 |  | Van et al 2013 |
|  | 0/177 |  | 0/179 |  |  | 8/532 |  | Van et al 2013 |
|  | 12/234 |  |  |  |  |  |  | Kirby et al 1998 |
|  | 6/196 |  |  |  |  |  |  | Roehrborn ＆ Siegel 1996 |
|  | 1/73 |  |  |  |  |  |  | Brawer et al 1993 |
|  | 17/501 |  |  |  |  |  |  | Lepor 1998 |
|  | 21/492 |  |  |  |  |  |  | Narayan ＆ Tewari 1998 |
|  | ***2.43%*** | ***0*** | ***0*** | ***3.69%*** |  | ***1.68%*** |  | ***overall*** |
| Dry mounth |  |  |  |  |  | 2/85 |  | Lee et al 2011 |
|  | 1/77 |  |  |  |  | 2/132 |  | Bae et al 2011 |
|  |  |  |  |  |  | 10/148 |  | Kaplan et al 2013 |
|  | 4/69 |  |  |  |  | 26/142 |  | Lee et al 2005 |
|  | 10/209 |  |  |  |  | 32/209 |  | MacDiarmid et al 2008 |
|  | 6/212 |  |  |  |  | 37/423 |  | Yamaguchi et al 2011 |
|  | 0/36 |  |  |  |  | 3/33 |  | Yang et al 2007 |
|  | 0/60 |  |  |  |  | 4/122 |  | Nishizawa et al 2011 |
|  | 1/326 |  |  |  |  | 61/661 |  | Van et al 2013 |
|  | 4/177 |  | 8/179 |  |  | 47/532 |  | Van et al 2013 |
|  | 11/251 |  |  |  |  |  |  | Jardin et al 1991 |
|  | ***2.61%*** |  | ***4.47%*** |  |  | ***9.01%*** |  | ***overall*** |
| Urinary retention |  |  |  |  |  | 1/85 |  | Lee et al 2011 |
|  | 2/358 | 1/344 |  |  | 1/349 |  |  | Debruyne et al 1998 |
|  | 0/212 |  |  |  |  | 4/423 |  | Yamaguchi et al 2011 |
|  | 0/60 |  |  |  |  | 1/122 |  | Nishizawa et al 2011 |
|  |  | 1/47 |  |  |  |  |  | Kirby et al 1992 |
|  | 1/326 |  |  |  |  | 5/661 |  | Van et al 2013 |
|  | 1/177 |  | 1/179 |  |  | 4/532 |  | Van et al 2013 |
|  | ***0.35%*** | ***0.51%*** | ***0.56%*** |  | ***0.29%*** | ***0.82%*** |  | ***overall*** |
| Micturtion difficulties | 1/77 |  |  |  |  | 6/132 |  | Bae et al 2011 |
|  | 1/69 |  |  |  |  | 4/142 |  | Lee et al 2005 |
|  | 0/60 |  |  |  |  | 1/122 |  | Nishizawa et al 2011 |
|  | ***0.97%*** |  |  |  |  | ***2.78%*** |  | ***overall*** |
| constipation |  |  |  |  |  | 50/148 |  | Kaplan et al 2013 |
|  | 0/69 |  |  |  |  | 3/142 |  | Lee et al 2005 |
|  | 5/212 |  |  |  |  | 30/423 |  | Yamaguchi et al 2011 |
|  | 0/60 |  |  |  |  | 3/122 |  | Nishizawa et al 2011 |
|  | 1/326 |  |  |  |  | 25/661 |  | Van et al 2013 |
|  | 2/177 |  | 4/179 |  |  | 14/532 |  | Van et al 2013 |
|  | 1/60 |  |  |  |  |  |  | Lloyd et al 1992 |
|  | ***1.00%*** |  | ***2.23%*** |  |  | ***6.16%*** |  | ***overall*** |
| hypotension | 8/358 | 3/344 |  |  | 2/349 |  |  | Debruyne et al 1998 |
|  | 2/470 |  |  |  |  |  |  | Nording 2005 |
|  | 22/1291 |  |  |  |  |  |  | Kirby et al 2001 |
|  | 4/81 |  |  |  |  |  |  | Elhilali et al 1996 |
|  | 3/293 |  |  |  |  |  |  | Van et al 2000 |
|  | 5/251 |  |  |  |  |  |  | Jardin et al 1991 |
|  | 8/196 |  |  |  |  |  |  | Roehrborn ＆ Siegel 1996 |
|  | 11/639 |  |  |  |  |  |  | Andersen et al 2000 |
|  | 0/198 |  |  |  |  |  |  | Fabricius et al 1990 |
|  | ***1.67%*** | ***0.87%*** |  |  | ***0.57%*** |  |  | ***overall*** |
| Sexual dysfunction | 8/358 | 23/344 |  |  | 26/349 |  |  | Debruyne et al 1998 |
|  | 6/196 | 7/204 |  |  |  |  |  | Rigatti et al 2003 |
|  | 0/103 | 5/102 |  |  |  |  |  | Lee 2002 |
|  | 11/470 |  |  |  |  |  |  | Nording 2005 |
|  | 7/335 |  |  |  |  |  |  | Roehrborn 2001 |
|  |  | 2/47 |  |  |  |  |  | Kirby et al 1992 |
|  | 2/177 |  | 1/179 |  |  | 2/532 |  | Van et al 2013 |
|  | 61/1611 | 97/1623 |  |  | 119/1610 |  |  | COMBAT |
|  |  | 0/126 |  |  |  |  |  | Na et al 2012 |
|  | 1/293 |  |  |  |  |  |  | Van et al 2000 |
|  | 6/73 |  |  |  |  |  |  | Brawer et al 1993 |
|  | ***2.82%*** | ***5.48%*** | ***0.56%*** |  | ***7.39%*** | ***0.38%*** |  | ***overall*** |
| Ejaculation disorders | 0/358 | 5/344 |  |  | 3/349 |  |  | Debruyne et al 1998 |
|  | 6/196 | 2/204 |  |  |  |  |  | Rigatti et al 2003 |
|  | 0/103 | 3/102 |  |  |  |  |  | Lee 2002 |
|  | 1/20 |  |  |  | 1/20 |  |  | Arora et al 2012 |
|  | 0/46 | 0/14 |  |  |  |  |  | Singh et al 2013 |
|  | 7/470 |  |  |  |  |  |  | Nording 2005 |
|  | 0/326 |  |  |  |  | 5/661 |  | Van et al 2013 |
|  | 0/177 |  | 0/179 |  |  | 1/532 |  | Van et al 2013 |
|  | 44/1611 | 23/1623 |  |  | 136/1610 |  |  | COMBAT |
|  | 7/198 |  |  |  |  |  |  | Fabricius et al 1990 |
|  | 59/501 |  |  |  |  |  |  | Lepor 1998 |
|  | 72/492 |  |  |  |  |  |  | Narayan ＆ Tewari 1998 |
|  | ***4.36%*** | ***1.44%*** | ***0*** |  | ***7.07%*** | ***0.50%*** |  | ***overall*** |
| Decreased libido | 2/358 | 6/344 |  |  | 7/349 |  |  | Debruyne et al 1998 |
|  | 0/103 | 4/102 |  |  |  |  |  | Lee 2002 |
|  | 0/20 |  |  |  | 1/20 |  |  | Arora et al 2012 |
|  | 1/46 | 0/18 |  |  |  |  |  | Singh et al 2013 |
|  | 27/1611 | 45/1623 |  |  | 55/1610 |  |  | COMBAT |
|  |  | 2/24 |  |  |  |  |  | Yu et al 1995 |
|  |  | 2/126 |  |  |  |  |  | Na et al 2012 |
|  | ***1.40%*** | ***2.64%*** |  |  | ***3.18%*** |  |  | ***overall*** |
| nasopharyngitis | 3/168 |  |  | 5/171 |  |  |  | Oelke et al 2012 |
|  | 1/103 | 0/102 |  |  |  |  |  | Lee 2002 |
|  | 1/152 |  |  | 5/306 |  |  |  | Yokoyama et al 2013 |
|  | 5/49 |  |  | 1/51 |  |  |  | Kim et al 2011 |
|  |  |  |  | 8/189 |  |  |  | McVary et al 2007 |
|  |  |  |  | 2/138 |  |  |  | McVary et al 2007 |
|  |  |  |  | 18/845 |  |  |  | Roehrborn et al 2008 |
|  |  |  |  | 12/544 |  |  |  | Brock et al 2013 |
|  | 7/335 |  |  |  |  |  |  | Roehrborn 2001 |
|  | 8/81 |  |  |  |  |  |  | Elhilali et al 1996 |
|  | 4/73 |  |  |  |  |  |  | Brawer et al 1993 |
|  | 88/501 |  |  |  |  |  |  | Lepor 1998 |
|  | 119/492 |  |  |  |  |  |  | Narayan ＆ Tewari 1998 |
|  | ***12.08%*** | ***0*** |  | ***2.27%*** |  |  |  | ***overall*** |
| Altered vision | 1/69 |  |  |  |  | 2/142 |  | Lee et al 2005 |
|  | 0/60 |  |  |  |  | 2/122 |  | Nishizawa et al 2011 |
|  | 0/177 |  | 2/179 |  |  | 3/532 |  | Van et al 2013 |
|  | ***0.32%*** |  | ***1.11%*** |  |  | ***0.88%*** |  | ***overall*** |
| myalgia | 0/152 |  |  | 9/306 |  |  |  | Yokoyama et al 2013 |
|  | 1/49 |  |  | 3/51 |  |  |  | Kim et al 2011 |
|  |  |  |  | 18/845 |  |  |  | Roehrborn et al 2008 |
|  |  |  |  | 7/544 |  |  |  | Brock et al 2013 |
|  | ***0.50%*** |  |  | ***2.12%*** |  |  |  | ***overall*** |
| Increased PVR | 2/212 |  |  |  |  | 19/423 |  | Yamaguchi et al 2011 |
|  | 0/60 |  |  |  |  | 1/122 |  | Nishizawa et al 2011 |
|  | 0.74% |  |  |  |  | 3.67% |  | overall |
| syncope | 3/470 |  |  |  |  |  |  | Nording 2005 |
|  | 0/81 |  |  |  |  |  |  | Elhilali et al 1996 |
|  | 1/198 |  |  |  |  |  |  | Fabricius et al 1990 |
|  | ***0.53%*** |  |  |  |  |  |  | ***overall*** |
| gynaecornastia | 23/1611 | 55/1623 |  |  | 58/1610 |  |  | COMBAT |
|  |  | 1/126 |  |  |  |  |  | Na et al 2012 |
|  | ***1.43%*** | ***3.20%*** |  |  | ***3.60%*** |  |  | ***overall*** |

Common reported adverse events are summarized stratified according to the treatment arms. An overall percentage (%) is reported at the bottom of each adverse event. AEs = adverse events. ABs = α-blockers. 5ARIs = 5α-reductase inhibitors. MRAs = muscarinic receptor antagonists. PDE5-Is = phosphodiesterase 5 inhibitors.
